# Three-dimensional-printed collagen/chitosan/secretome derived from HUCMSCs scaffolds for efficient neural network reconstruction in canines with traumatic brain injury

**DOI:** 10.1093/rb/rbac043

**Published:** 2022-06-27

**Authors:** Xiaoyin Liu, Guijun Zhang, Pan Wei, Lin Zhong, Yaxing Chen, Jianyong Zhang, Xuyi Chen, Liangxue Zhou

**Affiliations:** Department of Neurosurgery, West China Hospital, West China Medical School, Sichuan University, Chengdu 610041, Sichuan, China; Tianjin Key Laboratory of Neurotrauma Repair, Pingjin Hospital Brain Center, Characteristic Medical Center of People's Armed Police Forces, Tianjin 300162, China; Department of Neurosurgery, West China Hospital, West China Medical School, Sichuan University, Chengdu 610041, Sichuan, China; Department of Neurosurgery, The First People's Hospital Of Long Quan yi District, Chengdu 610000, Sichuan, China; The First Affiliated Hospital of Chengdu Medical College, Chengdu 610500, Sichuan, China; Department of Neurosurgery, West China Hospital, West China Medical School, Sichuan University, Chengdu 610041, Sichuan, China; Department of General Surgery, the Affiliated Hospital of Guizhou Medical University, Guiyang CN 540000, P. R., Guizhou, China; Tianjin Key Laboratory of Neurotrauma Repair, Pingjin Hospital Brain Center, Characteristic Medical Center of People's Armed Police Forces, Tianjin 300162, China; Institute of Medical Security for Maritime Rights Protection of Characteristic Medical Center of Chinese People's Armed Police Force (PAP), Tianjin, 300162, China; Department of Neurosurgery, West China Hospital, West China Medical School, Sichuan University, Chengdu 610041, Sichuan, China

**Keywords:** traumatic brain injury, canines, secretome, low temperature extrusion 3D printing, collagen, chitosan

## Abstract

The secretome secreted by stem cells and bioactive material has emerged as a promising therapeutic choice for traumatic brain injury (TBI). We aimed to determine the effect of 3D-printed collagen/chitosan/secretome derived from human umbilical cord blood mesenchymal stem cells scaffolds (3D-CC-ST) on the injured tissue regeneration process. 3D-CC-ST was performed using 3D printing technology at a low temperature (−20°C), and the physical properties and degeneration rate were measured. The utilization of low temperature contributed to a higher cytocompatibility of fabricating porous 3D architectures that provide a homogeneous distribution of cells. Immediately after the establishment of the canine TBI model, 3D-CC-ST and 3D-CC (3D-printed collagen/chitosan scaffolds) were implanted into the cavity of TBI. Following implantation of scaffolds, neurological examination and motor evoked potential detection were performed to analyze locomotor function recovery. Histological and immunofluorescence staining were performed to evaluate neuro-regeneration. The group treated with 3D-CC-ST had good performance of behavior functions. Implanting 3D-CC-ST significantly reduced the cavity area, facilitated the regeneration of nerve fibers and vessel reconstruction, and promoted endogenous neuronal differentiation and synapse formation after TBI. The implantation of 3D-CC-ST also markedly reduced cell apoptosis and regulated the level of systemic inflammatory factors after TBI.

## Graphical Abstract

**Figure:**
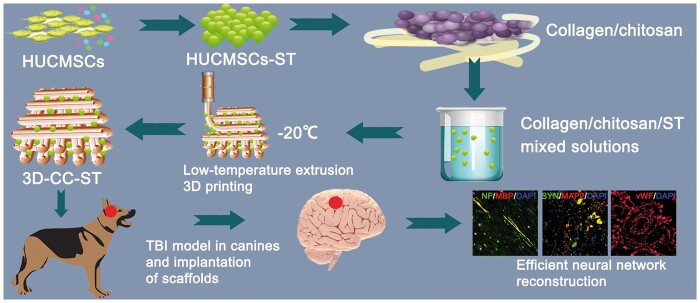

## Introduction

Traumatic brain injury (TBI) is classified into mild, moderate and severe injuries, leading to a duration of acute (days to weeks) or chronic (months to years) symptoms, respectively, which disrupt normal brain function, resulting in physical and emotional distoration [1–4]. The incidence rate increases sharply with age; every year, 50 million new mild TBI cases and 2 million new middle/severe TBI cases are observed worldwide [5]. Approximately 53.17% of patients with TBI in China suffer a poor prognosis with permanent disability at the endpoint [6]. Undoubtedly, TBI poses a great challenge in health recovery for patients. With advances in medical technology in recent years, various TBI management strategies have been proposed and performed. Unfortunately, no available and defined therapeutic method has been recommended to confer a benefit to functional recovery for TBI patients in clinical practice.

Stem cells have emerged as a promising therapeutic choice and are the focus in the area of regenerative medicine. The different types of human pluripotent stem cells are embryonic stem cells, adult stem cells, and induced pluripotent stem cells. They have the ability to self-renew and differentiate into a diversity of cell types, which makes it possible to use cell therapy for irreversible injury [7]. Mesenchymal stem cells (MSCs) can be isolated from multiple tissues, such as teeth, umbilical cord and bone marrow. Of these, umbilical cord-derived MSCs (UCMSCs) have the best potential and development capacity, without invasive separation at birth and moral as well as spiritual barriers for isolation [8]. MSC transplantation in TBI models is considered one of the most promising therapeutic strategies for anti-inflammation, impeding the cavity information and neuro-regeneration [9–11].

The mechanism by which stem cells implement their therapeutic effects is well understood. Accumulating literature advocates that the secretome (ST) of MSCs, including various bioactive factors, contributes to the therapeutic merits of MSCs, which is considered a promising and encouraging replacement of corresponding cells for immune regulators [12]. These factors mainly consist of soluble factors (growth factors, cytokines and chemokines) and extracellular vesicles (microvesicles and exosomes), which can regulate neuroinflammation, leading to a favorable neural functional outcome [13].

On the other hand, increasing evidence shows that the creation of 3D-printed scaffolds bridges the bifurcation between the engineered tissue and native tissue, which not only serves as a template in which new tissue can be generated, but also supports the exchange of oxygen and nutrients [14, 15]. Synthetic polymers and naturally derived materials are two main biomaterials [16]. The former usually lack bioactivity, and because of this, its degradation can cause tissue necrosis [17]. In contrast, chitosan, gelatin and hyaluronan usually exhibit better cell adhesion, culture, growth and differentiation [18, 19]. Collagen represents an insoluble fibrous protein, the major component of the extracellular matrix, attracting scholars’ attention owing to its advantage of stiffness and integrity, which are equipment with an excellent delivery system for bioactive molecules or drugs [5, 20–22]. Chitosan, a functional protein with excellent biocompatibility, biodegradability and biosafety, is also a suitable candidate for biomedical and tissue engineering fields [23, 24]. While it is insoluble in a neural solution with a pH ≥ 7, chitosan can be deposited from an acid solution [25]. Recently, collagen/chitosan composites have been used extensively in tissue engineering, notably in bone regeneration, wound healing and TBI [26–28]. However, there are still some difficulties that fail to connect or fill the anatomic structure of the lost brain tissue. Biomaterials based on 3D printing technologies can solve these problems by tailoring size and suitable control over the microenvironment [29]. However, 3D-printed scaffolds based on the printing technique, usually with an extremely high temperature, will cause the loss of growth factor bioactivity, leading to a bioefficacy impairment of such sensitive growth regulators.

Inspired by traditional limitations, in this study, we comprehensively estimated and compared the effect of 3D-printed collagen/chitosan scaffolds (3D-CC) and 3D-printed collagen/chitosan/secretome derived from human umbilical cord blood MSCs (HUCMSCs) scaffolds (3D-CC-ST) on cell adhesion and proliferation *in vitro*. In addition, a new method for placing secretomes derived from HUCMSCs (HUCMSCs-ST) into this scaffold at a low temperature during the 3D printing process was described. To provide a more in-depth understanding of the injured tissue regeneration process, a detailed evaluation was performed using the *in vivo* canine model assay system.

## Materials and methods

### Culture of HUCMSCs and isolation of HUCMSCs-ST

We have previously described a method for the isolation and culture of HUCMSCs [30, 31]. Generally, umbilical cord blood samples were taken from pregnant women in the obstetrics and gynecology department of Characteristic Medical Center of People’s Armed Police Forces and then subjected to HUCMSC separation based on density gradient centrifugation and the direct adherence method. And then it was placed in an incubator with DMEM supplemented with 20% PBS, 2 mM glutamine and antibiotics at 37°C with 5% CO_2_.

HUCMSCs-ST was collected in accordance with previous report [13]. Generally, to obtain adequate ST, the primary HUCMSCs were seeded onto 75 cm^2^ culture flasks and maintained in DMEM supplemented with 10% fetal bovine serum for 24 h. Then the medium was replaced with serum-free, low-glucose DMEM. After 24 h, the conditioned medium was collected in serum-free DMEM with low glucose. Then, following centrifugation at 500 × g for 10 min, the media was centrifuged at 800 × g for 15 min twice at 4°C, and a Minimate TFF capsule system (PALL Corporation, Ann Arbor, MI, USA) with a 100-kDa membrane was used to concentrate the supernatants by ultrafiltration. Finally, the ST was concentrated to 20 μl at a number of 1 × 10^7^ cells. The protein concentration of ST was calculated by a bicinchoninic acid (BCA) protein assay kit (Beyotime, China). A cytokine array system (RayBio Human Cytokine Antibody Array C Series 2000; RayBiotech Inc. Norcross, GA, USA) was used to detect specific proteins in the supernatant of HUCMSCs according to published literature [13, 32].

### Fabrication of 3D-printed scaffolds

The fabrication of this scaffold has been described in a previous study [33, 34]. Generally, pulverized fresh bovine tend was completely crushed and then immersed in 0.05 mmol/l Tris buffer to remove soluble impurities. After centrifugation, we collected the precipitate, which was dissolved in an acetic acid solution (Solarbio Science & Technology Co., Ltd, China) containing pepsin (Aladdin Biotechnology Co., Ltd, Shanghai, China) with sufficient dissolution, and the supernatant was obtained by centrifugation. A 3.5 mol/l NaCl solution was added to the collected salt precipitate; consequently, collagen gel was acquired following dialization in 4°C deionized water over 5 days. The collagen and chitosan (deacetylation degree 75–85%, Sigma, USA) were dissolved in 1% acetic acid solution in a certain ratio of 1:8.

In terms of 3D printing, serial compound materials first need to be incubated overnight at 4°C. Prior to the beginning, notably, to ensure the sufficiently even distribution of ST in the scaffold, 0.1 g of serial compound material was soaked with 20 μl ST of solution (200 μg) and incubated for 24 h at 4°C. The mixed solution was incubated overnight at 4°C, followed by stirring at 4°C for 12 h. Subsequently, constructs were ready for 3D printing at 4°C. The following parameters were used: (i) platform temperature at −20°C; (ii) nozzle diameter at 160 μm; (iii) extension speed at 0.17 mm/min; (iv) printing speed at 12 mm/s and (v) thickness at 0.3 mm/layer. Eventually, the mixed solution (collagen/chitosan compound materials with HUCMSCs-ST or collagen/chitosan compound material alone) was ready to be placed in a printer cartridge. After printing, the 3D solid model (3D-CC-ST or 3D-CC), followed by vacuum cooling and drying for 48–72 h, was stored overnight at −80°C. Cylindrical scaffolds with a diameter of 2 mm and a height of 2 mm were formed by cutting. These scaffolds were based on our design and divided into five different scaffolds: the collagen scaffolds (C) group, the collagen/chitosan scaffolds (CC) group, the 3D-CC group, the 3D-printed collagen scaffolds (3D-C) group and the 3D-CC-ST group. 3D-CC-ST were performed at low temperature to sustain the bioactivity of HUCMSC-ST.

### *In vivo* degradation test

An *in vivo* degradation test was performed according to a previous study [35]. Twenty-four female Sprague–Dawley (SD) rats, 2 months old and weighing ∼200 g, were obtained. 3D-CC-ST with 5 five mass ratios (collagen: chitosan = 1:1, 1:4, 1:8, 1:12, 1:16) were designed separately, and we recorded the initial weight. Three small openings of 1 cm were performed, following an intraperitoneal injection of 1% pentobarbital sodium for anesthesia. Three sterile 3D-CC-ST with the same content ratio were implanted in a rat with three incisions. Then, the scaffolds were carried out at 1, 2, 3, 4, 5 and 6 months after surgery. The enzyme solution was removed and the scaffold was decellularized to determine the remaining dry weight. The degradation rate of the scaffold was calculated as: percent mass remaining = (mass at time (*t*)/initial mass) × 100%.

### Scaffold characteristics

For the porosity ratio, the scaffolds were placed in absolute ethanol. The volume of ethanol (V1) was not recorded until a negative pressure dressing with no bubbles had been down. The volume of ethanol (V2), excluding the scaffold, was recorded as V3. The porosity ratio was calculated by the formula: porosity ratio (%) = (V1 − V3)/(V2 − V3) × 100%. For the water absorption ratio, the three dried samples were taken out and placed for 24 h in a 0.01 mol/l PBS buffer solution (pH = 7.4) before the measurements were carried out [34]. The water absorption ratio was calculated by measuring the weights of the dry (m1) after drying in the drying cabinet for 2 h and absorbing moisture (m0). The water absorption was calculated using the equation: water absorption (100%) = (m0 − m1)/m1 × 100%. For mechanical strength, the compressive strength of 3D-printed scaffold constructs was determined using an Instron 5865 machine (Instron, Norwood, NA, USA). The related parameters were set before biomechanical loading.

### ST releases from 3D-CC-ST

The BCA kit was used to examine the secretome release and cumulative secretome release from 3D-CC-ST according to a previous report [36, 37]. Immediately after scaffolds preparation, 3D-CC-ST were washed with PBS to remove the free secretome. 3D-CC-ST was then soaked in PBS. Supernatants were collected at 1, 4, 7, 14, 21 and 28 days after soaking, and a BCA kit was used to detect the amount of free secretome in the supernatants.

### Scaffold cytocompatibility evaluation

With regard to the cell viability assay, the third generation of HUCMSCs at a concentration of 1 × 10^6^/l (100 μl) with a pipette were seeded on 3D-CC and 3D-CC-ST. At 7 days after coculture, inverted phase-contrast microscopy (Nikon, Tokyo, Japan), scanning electron microscopy (SEM) (Hitachi, Tokyo, Japan), and hematoxylin and eosin (HE) staining were performed to observe the growth of cells on these two types of scaffolds. Cell proliferation in 3D bioprinting constructs was evaluated by the MTT assay (Solarbio Science & Technology Co., Ltd). The values in both groups were shown in a time-dependent manner at 1, 3, 5 and 7 days after coculture.

### TBI model in canines and implantation of scaffolds

Twenty male canines were prepared in this study and were randomly divided into four groups: the Sham group (only the skull was opened without TBI, *n* = 5), the TBI group (TBI without any implantation, *n* = 5), the 3D-CC group (TBI with the implantation of 3D-printed collagen/chitosan scaffolds, *n* = 5) and the 3D-CC-ST group (TBI with the implantation of 3D-printed collagen/chitosan/secretome derived from human umbilical cord blood MSCs scaffolds, *n* = 5). The TBI model was described in a previous study [38]. After a sequence of lesions were made on the scalp, bone and dural mater, an injury was performed in the right cerebral hemisphere based on a modified electric cortical contusion impactor: the parameters were as follows: 9.99 mm in depth, 5.34/s in speed and 255 m/s in dwell time. Immediately after the establishment of the TBI model, 3D-CC-ST and 3D-CC were implanted into the TBI lesion area.

### Neurological examination

Neurological function deficits in canines were examined and screened on a modified Galasne score system (mGCS) initiated by Platt *et al*. [39] as follows: a score of 3 suggests brain damage and a score of 18 suggests health; a score initiated by Purdy *et al.* [40] as follows: a score of 2 suggests health and a score of 11 suggests coma or death, and an NDS scale initiated by Castellá *et al*. [41] as follows: a score of 0 suggests health and a score of 500 suggests brain damage. At 1 day, 1, 2, 4, 8, 16, 20 and 24 weeks after surgery, all animals were tested by two authors blinded to this study (*n* = 5 for each group).

### Motor evoked potential detection

Electrophysological assays were performed to obtain motor evoked potential (MEP) at 6 months after surgery by using an evoked potential meter (VIKING QUEST4, Thermo Nicolet Corporation, USA) (*n* = 5 for each group). Changes in the amplitude and latency of MEP of the four limbs were determined. The parameters of electrophysiological analysis were as follows: stimulation voltage = 90 V, pulse width = 0.5 m/s and stimulation frequency = 500 Hz. This determination was previously described in the literature [35, 42]. Generally, stimulating electrodes are made by attaching two needles to the cranial muscle. The detection electrodes are made by inserting two needles into each muscle of the extremity. The ground wire is made by attaching an electrode to the tail.

### HE, Bielschowsky’s silver, Nissl and Masson staining of brain tissue

At 6 months after TBI, the brain tissue was fixed in 10% formalin after being separated, and the samples were cut into 2 mm-thick slices across the lesion after being dehydrated by ethanol and xylene, and then embedded in paraffin. Generally, for HE, the sections were incubated in hematoxylin staining and washed in running tap water (*n* = 5 for each group). After rinsing with distilled water, the sections were placed in eosin. Then, they were dehydrated in ethanol and xylene and cover slipped. Bielschowsky’s silver staining was performed to ascertain nerve regeneration (*n* = 5 for each group). First, the sections were dewaxed in xylene, washed with distilled water, and then incubated in silver nitrate solution (Sigma, St Louis, MO, USA) in the dark for half an hour. They were placed in distilled water and formaldehyde solution and subsequently placed into a wet box before dark brown. Then, they were transferred into sodium sulfate (Sigma, St Louis, MO, USA) solution (5%). For Nissl staining, brain sections were collected and stained with 0.3% cresyl violet to assess neuronal density and the overall brain morphology of the injured brain (*n* = 5 for each group). Masson staining (MT) was performed after dewaxing in xylene, dehydrating in gradient alcohol and rinsing in distilled water (*n* = 5 for each group). The section was placed in hematoxylin, staining solution and then in phosphomolybdic acid solution and rinsed with acetic acid solution. All quantitative analyses of tissue staining were performed using ImageJ software.

### Immunofluorescence staining

At 6 months after TBI, immunofluorescence staining was used to quantify neural and vascular regeneration (*n* = 5 for each group). At designated time points, the samples were dewaxed and a solution of EDTA was added to repair the antigen. The slices were then treated with 5% donkey serum in PBS to minimize non-specific antibody binding, and incubated with purified primary antibodies (vWF: rabbit anti, 1:1000, Abcam, Cambridge, UK; NF: rabbit anti, 1:1000, Proteintech, USA; MBP: rat anti, 1:1000, Abcam, Cambridge, UK; GAP43: rabbit anti, 1:200, Abcam, Cambridge, UK; Tuj-1: rabbit anti, 1:400, Millipore, USA; SYN: rabbit anti, 1:1000, Bioss, Beijing, China; MAP2: chicken anti, 1:1000, Abcam, Cambridge, UK; PSD95: rabbit anti, 1:500, Abcam, Cambridge, UK) at 4°C overnight. Next, cell nuclei were stained with DAPI at room temperature. All images were taken under a fluorescence microscope (Leica TCS SP5, Germany). All quantitative analyses of immunofluorescence staining were performed using ImageJ software.

### TUNEL staining

At 6 months after TBI, the apoptosis rate of cells was detected by TUNEL staining (Promega Corporation) according to a previous study [43]. To analyze apoptosis rates in tissues, five regions were randomly selected for quantification of TUNEL-stained cells (*n* = 5 for each group). ImageJ software was performed to obtain TUNEL-positive cells per field.

### Measurement of plasma inflammatory factors using enzyme-linked immunosorbent assay

The expression levels of TNF-α, IL-6 and IL-10 around the brain injury, within a week and at 6 months after transplantation, were measured using customized enzyme-linked immunosorbent assay (ELISA) kits (Wuhan Servicebio Technology Co., Ltd, China) (*n* = 5 for each group). The cytokine levels in the tissue homogenates were normalized using the protein concentration in the samples.

### Statistical analysis

All the experimental data from at least three samples are expressed as the mean ± standard deviation (SD). Significant differences among groups were analyzed by one-way analysis. *P *<* *0.05 was considered significant, <0.05. ^##^*P *<* *0.01, ^#^*P *<* *0.05, **P <* *0.01 and *P <* *0.05.

## Results

### Morphology and microstructure of 3D-CC-ST

**Figure 1.**
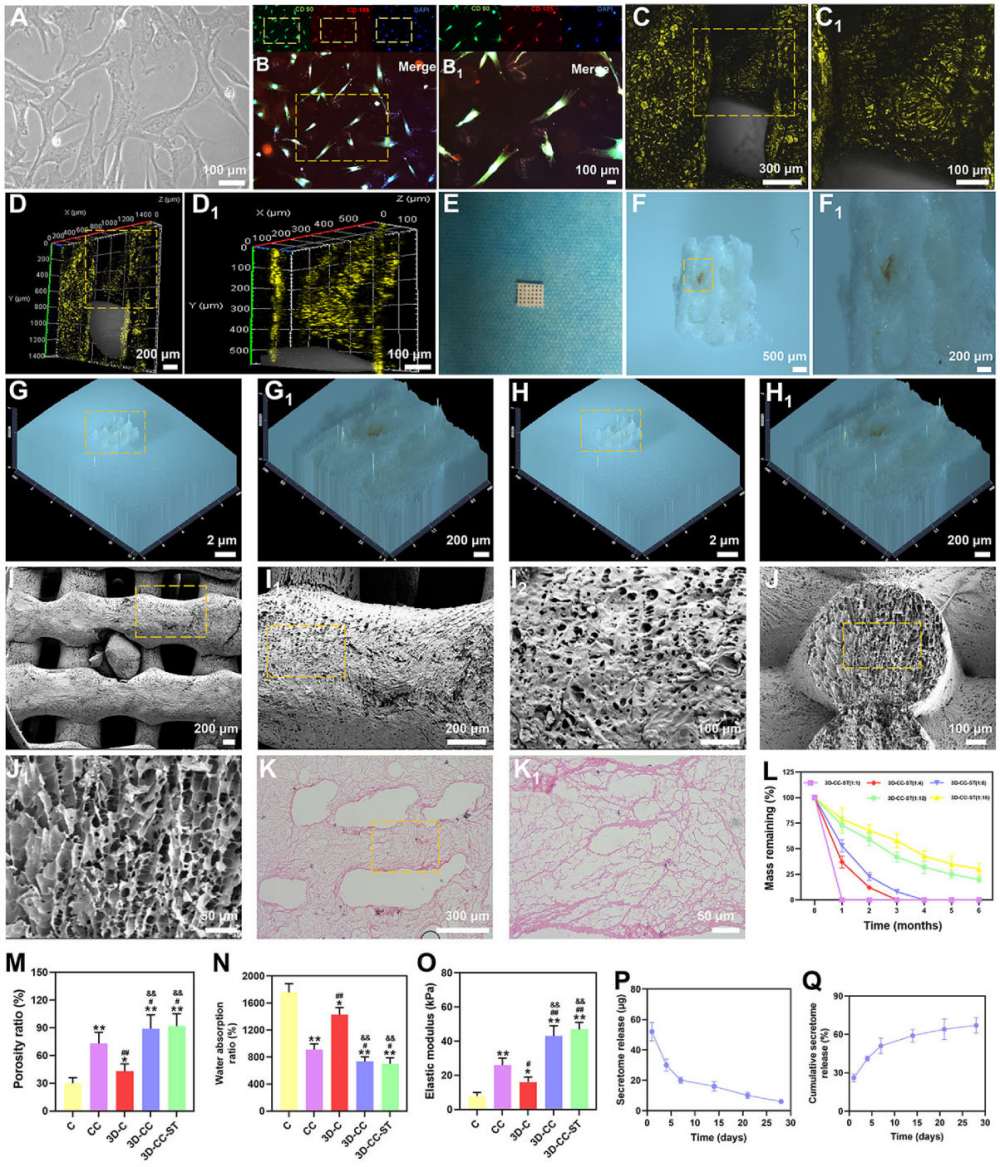
Characterization of HUCMSCs. Phase-contrast microscope (**A**). Immunofluorescence images of the biomarkers CD90 (green) and CD105 (red) in HUCMSCs (**B** and **B1**). Characterization of 3D-CC-ST. Representative morphological images of 3D-CC-ST under a confocal laser scanning microscope (**C**–**D1**), stereomicroscope (**F**–**H1**), SEM (**I**–**J1**) and HE (**K** and **K1**). Degradation curve of 3D-CC-ST with 5 five mass ratios (**L**). Porosity ratio (**M**), water absorption (**N**) and elastic modulus (**O**) for the five groups of scaffolds. Secretome release (**P**) and cumulative secretome release (**Q**) from 3D-CC-ST. *P <* *0.05, **P <* *0.01 vs C. ^#^*P *<* *0.05, ^##^*P *<* *0.01 vs CC. ^&&^*P *<* *0.01 vs 3D-C.

Phase-contrast microscopy (Fig. 1A) showed that fibroblastic cells were observed after the third passage, and cells derived from the HUCMSCs were positive for the surface antigens CD90 and CD105 (>95%) (Fig. 1B and B1), identifying mesenchymal features. Cytokine antibody array analysis revealed that HUCMSCs–ST contained 79 proteins. Seventy-nine proteins of HUCMSCs–ST have been shown in previous studies [13].

We investigated the morphological features of the 3D-printed scaffold under a confocal laser scanning microscope, stereomicroscope, SEM and HE, and the results are shown in Fig. 1C–K1. The results showed a rough face and porous structure, which is suitable for cell adhesion.

The degradation of all 3D-CC-ST gradually decreased with time and five different portions of collagen and chitosan (1:2, 1:4, 1:8, 1:12 and 1:16) were used to assess the optimal one (Fig. 1L). 3D-CC-ST with ratios of 1:1 and 1:4 showed an accelerated degradation rate within 3 months compared with the other three scaffolds. On the other hand, at 6 months after transplantation, 3D-CC-ST with a ratio of 1:12 and 1:16 remained more than 20% of its initial mass. At 6 months after transplantation, 3D-CC-ST with a ratio of 1:8 completely degraded. Taken together the results supported that the scaffold with a ratio of 1:8 played a major role in the degradation process. Therefore, 3D-CC-ST with a mass ratio of 1:8 was chosen for subsequent experiments.

3D-CC and 3D-CC-ST had a significantly higher porosity ratio than the other 3 groups (*P *<* *0.05) (Fig. 1M). In addition, the water absorption ratio of 3D-CC and 3D-CC-ST was significantly lower than that of the other 3 groups (*P *<* *0.05), promoting the maintenance of ST (Fig. 1N). Additionally, the elastic modulus of 3D-CC and 3D-CC-ST was significantly higher than that of the other three groups (*P *<* *0.01) (Fig. 1O), supporting better tissue regeneration. The HUCMSC-ST release from 3D-CC-ST was detected to assess the effect of binding on growth factors. The results of the secretome release and cumulative secretome release curve showed that the ST release from 3D-CC-ST lasted for more than 14 days, and more than 50% of the ST was released from 3D-CC-ST, which was beneficial to the biological effect on brain tissue (Fig. 1P and Q).

### 3D-CC-ST possess favorable cytocompatibility *in vitro*

**Figure 2.**
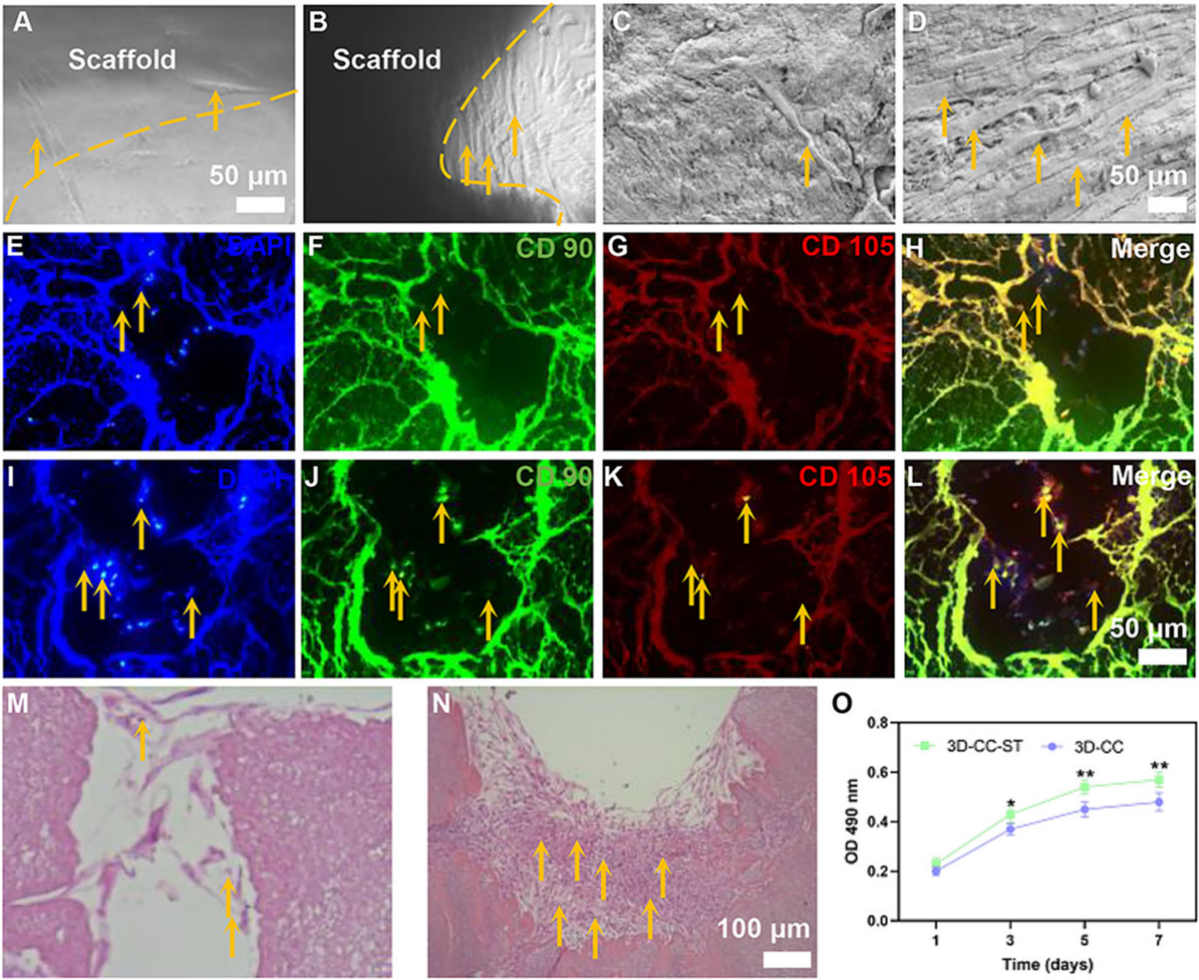
Cytocompatibility of the scaffold (**A**–**O**). The effects of 3D-CC (A, C, E–H, M) and 3D-CC-ST (B, D, I–L, N) scaffolds on the adhesion and proliferation of HUCMSCs under the SEM images (A–D) (yellow arrow), immunofluorescence staining images (E–L) (yellow arrow) and HE staining images (M–N) (yellow arrow). MTT assay of HUCMSCs cultured on 3D-CC and 3D-CC-ST scaffolds at 1, 3, 5 and 7 days (O). *P <* *0.05, **P <* *0.01 vs 3D-CC.

We studied the influence of the 3D-CC scaffold with/without ST on HUCMSCs. The patterns on these two scaffolds were satisfactory with the *in vitro* culture due to their suitable and uniform size. Both scaffolds indicated that they had the capacity to sustain HUCMSCs with an even distribution throughout the structure. Images of phase-contrast microscopy (Fig. 2A and B) and SEM (Fig. 2C and D) revealed that the number of fibroblasts that proliferated on 3D-CC-ST was higher than that on 3D-CC. Images of immunofluorescence staining (Fig. 2E–L) and HE staining (Fig. 2M and N) suggested that the number of cells attached to the 3D-CC-ST was favorably increased compared with that attached to the 3D-CC. The MTT assay was used to accurately evaluate cell proliferation at 1, 3, 5 and 7 days after seeding on the scaffold (Fig. 2O). The OD values of these two groups increased over time, with a significantly higher OD value for the 3D-CC-ST group than for the 3D-CC group (*P *<* *0.05). These results all indicated that 3D-CC-ST was more favorable for cell proliferation and adhesion of HUCMSCs than 3D-CC, possibly due to the addition of HUCMSCs-ST.

### Implanting 3D-printed collagen/chitosan/HUCMSCs-secretome scaffolds significantly ameliorated neurological function scores and MEP after TBI

**Figure 3.**
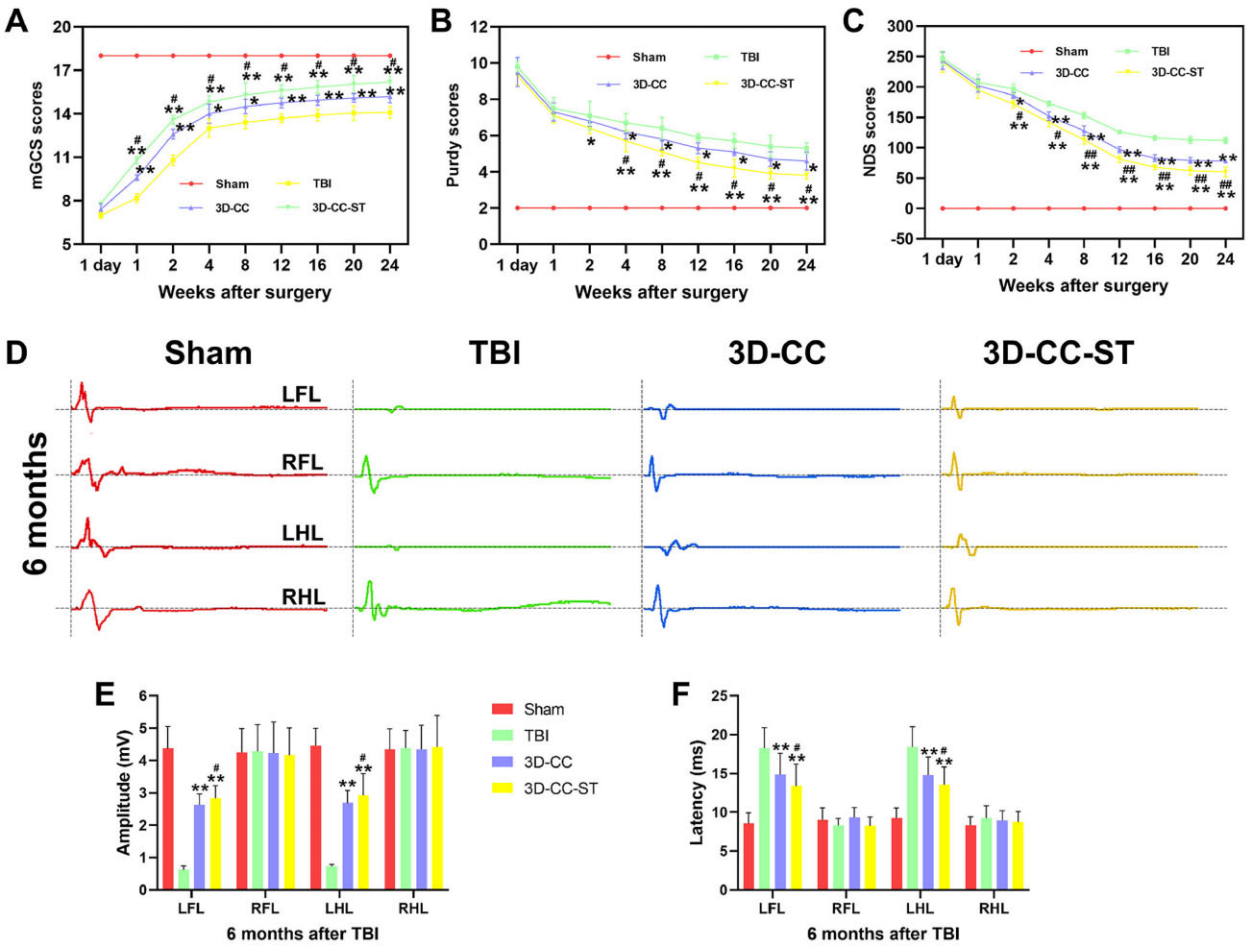
Locomotor function assessment and electrophysiological analysis of the four groups after TBI. mGCS (**A**), purdy (**B**) and NDS (**C**) scores at 1 day, 1, 2, 4, 8, 12, 16, 20 and 24 weeks after TBI. (**D**) Typical MEP schematic diagrams of the left forelimb (LFL), right forelimb (RFL), left hindlimb (LHL), and right hindlimb (RHL) at 6 months after TBI. Quantitative analysis of amplitude (**E**) and latency (**F**) of MEP at 6 months after TBI. *P <* *0.05, **P <* *0.01 vs TBI. ^#^*P *<* *0.05, ^##^*P *<* *0.01 vs 3D-CC.

Locomotor function was evaluated at 1 day, 1, 2, 4, 8, 12, 16, 20 and 24 weeks after TBI, in accordance with the mGCS, NDS and Purdy scores (Fig. 3A–C). Each group showed the same baseline prior to the injury, and displayed a similar neurological impairment at 1 day after TBI, excluding a slight functional recovery in the treatment group at 1 week by the mGCS score. Generally, compared with the restoration in the TBI group and 3D-CC group, a more rapid recovery of locomotor function in the treatment group was displayed. Significant differences in mGCS, NDS and Purdy scores were detected in the 3D-CC-ST group compared with the TBI group and 3D-CC group at each time point after 4 weeks (*P *<* *0.05).

At 6 months post-operation, the MEP showed an improvement in the amplitude and latency of the left forelimb and left hindlimb in the TBI group, the 3D-CC group and the 3D-CC-ST group (Fig. 3D). At 6 months after surgery, the 3D-CC-ST group significantly ameliorated the amplitude and latency of the left forelimb and left hindlimb compared with the TBI group and the 3D-CC group (*P *<* *0.05) (Fig. 3D–F).

### The implantation of 3D-printed collagen/chitosan/HUCMSCs-secretome scaffolds markedly reduced the cavity area, inhibited the formation of glial scars and facilitated vessel reconstruction after TBI

**Figure 4.**
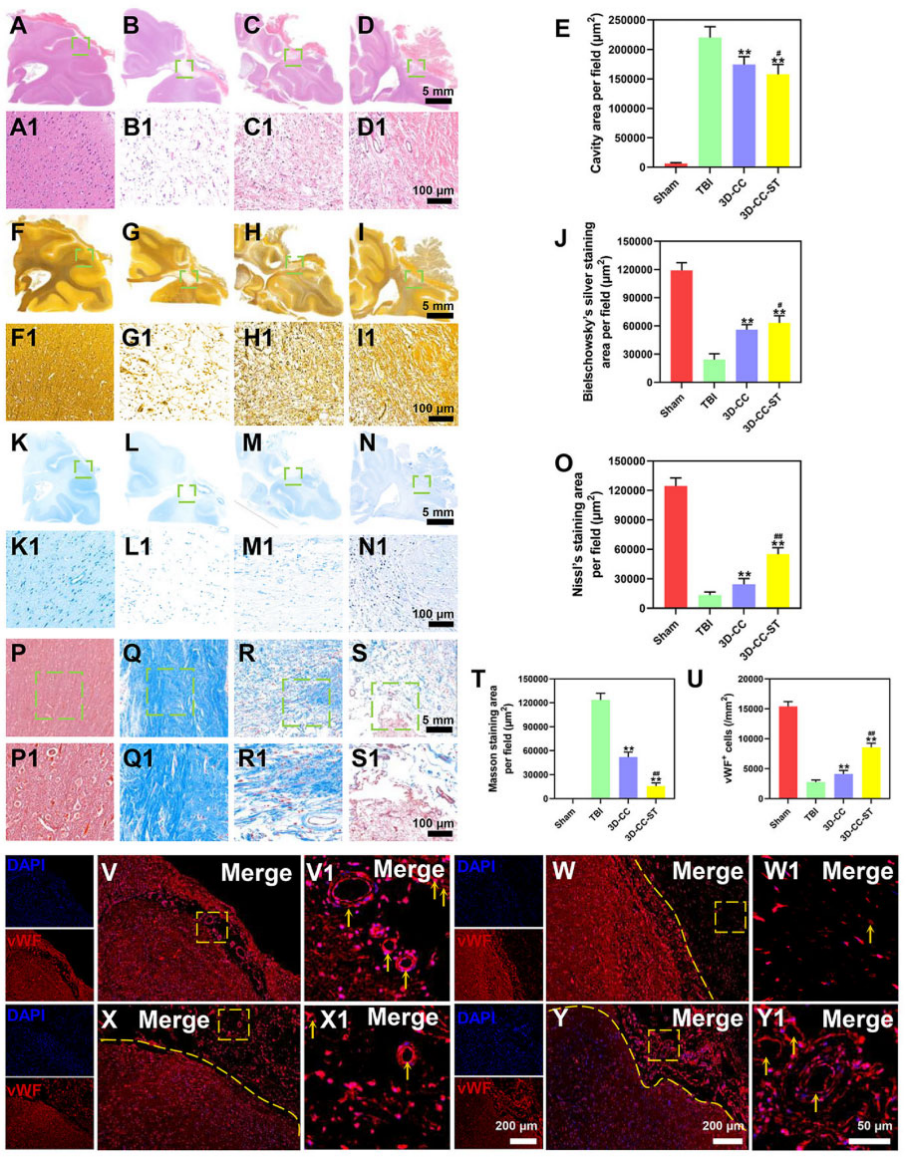
Histological analysis of brain tissue formation and vessel reconstruction around and within the scaffolds at month 6 post-surgery in the four groups. (**A**–**T**) HE staining (A–E), Bielschowsky’s silver staining (F–J), Nissl staining (K–O), Masson staining (P–T). (**V**–**U**) Immunostaining with vWF (red) at 6 months after TBI for the four groups: Sham group (V and **V1**), TBI group (**W** and **W1**), 3D-CC group (**X** and **X1**), and 3D-CC-ST group (**Y** and **Y1**). There were a few regenerated blood vessels and few vWF-positive cells inside the TBI group and 3D-C/C group (W and **X1**), while a larger number of regenerating vessels and many vWF-positive cells were detected in the 3D-C/C+ST group (Y and Y1). Quantification of regenerated brain tissue and vessel reconstruction in the peri-injured area after TBI (E, J, O, T and U). **P <* *0.01 vs TBI. ^#^*P *<* *0.05, ^##^*P *<* *0.01 vs 3D-CC.

HE staining revealed that implanting 3D-CC-ST significantly reduced the cavity area compared with implanting 3D-CC and/or without implanting scaffolds (*P *<* *0.05) (Fig. 4A–E). We examined the degree of nerve fiber regeneration in the trauma foci with Bielschowsky’s silver staining (Fig. 4F–J). The injured tissue displayed a reduction in nerve fibers in the TBI group and the 3D-CC group, whereas in the 3D-CC-ST group, massive regeneration fibers were detected (*P *<* *0.05) (Fig. 4F–J). The number of dead neuronal cells on Nissl staining in the TBI group was found in the lesions of the cerebral location (Fig. 4K–O). Numerous surrounding neuronal cells were observed in the 3D-CC-ST group. A significant increase in the number of neurons was detected in the 3D-CC-ST group compared with the TBI group and the 3D-CC group, indicating that treatment with based 3D-CC-ST conferred a neuronal survival benefit (*P *<* *0.01) (Fig. 4K–O). Masson staining revealed that increased glial scars migrated to the injury site in the TBI group and the 3D-CC group, while few fibers were detected in the 3D-CC-ST group (Fig. 4P–T) (*P *<* *0.01). The influence of the selected candidate protein vWF on vascular regeneration was investigated (Fig. 4V and U). The 3D-CC-ST group showed more positive expression of vWF than the TBI group and the 3D-CC group, indicating more vessel reconstruction in the 3D-CC-ST group (*P *<* *0.01).

### Implanting 3D-printed collagen/chitosan/HUCMSCs-secretome scaffolds significantly facilitated the regeneration of nerve fibers and axons and enhanced remyelination after TBI

**Figure 5.**
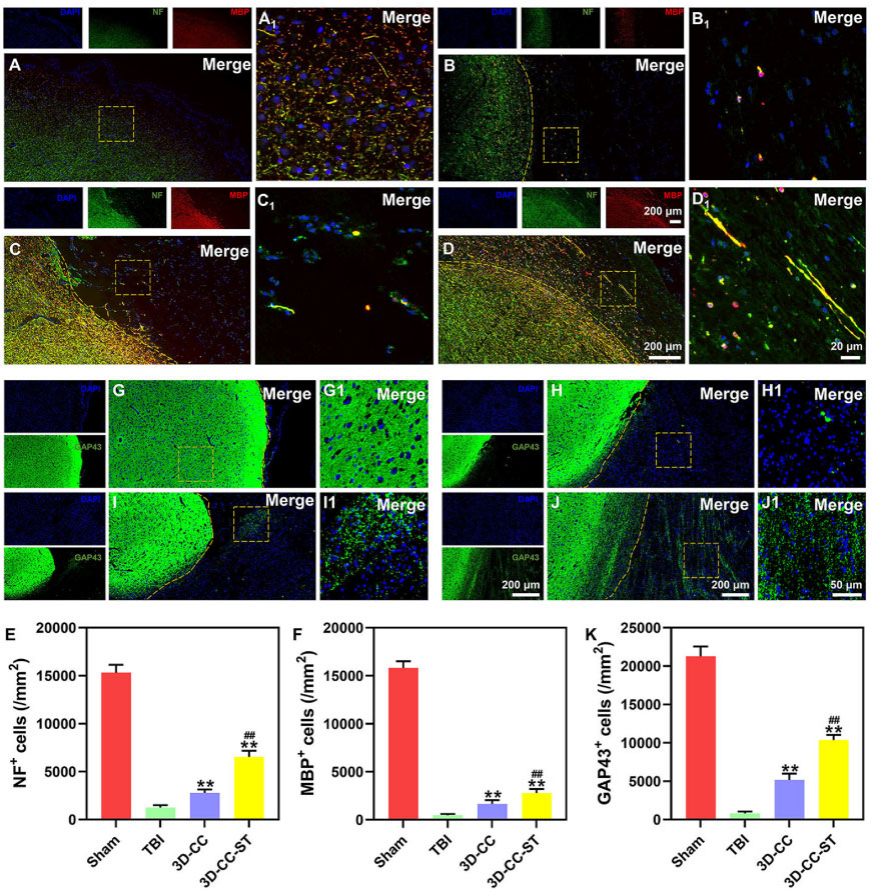
Regeneration of nerve fibers, myelin sheaths and axons *in vivo* at 6 months after TBI. Expression of NF (green) and MBP (red) in the four groups: Sham group (**A** and **A1**), TBI group (**B** and **B1**), 3D-CC group (**C** and **C1**) and 3D-CC-ST group (**D** and **D1**). The number of NF- and MBP-positive cells in the 3D-CC-ST group was significantly increased compared with that in the TBI group and the 3D-CC group. Quantification of NF- and MBP-positive cells in the peri-injured area after TBI (**E** and **F**). Expression of GAP43 (green) in the four groups: Sham group (**G** and **G1**), TBI group (**H** and **H1**), 3D-C/S group (**I** and **I1**) and 3D-CC-ST group (**J** and **J1**). The 3D-CC-ST group showed significantly more positive cells of GAP43 than the TBI group and the 3D-CC group. Quantification of GAP43-positive cells in the peri-injured area after TBI (**K**). **P <* *0.01 vs TBI; ^##^*P *<* *0.01 vs 3D-CC.

We used NF, MBP and GAP43 as indicators of nerve fibers, myelin sheaths and axons. More positive cells expressed NF and MBP in the 3D-CC-ST group than in the TBI group and the 3D-CC group (*P *<* *0.01) (Fig. 5A–F). GAP43-positive cells were expressed in the 3D-CC-ST group, whereas few corresponding cells were expressed in the TBI group and the 3D-CC group. These results suggested that 3D-CC-ST could facilitate the regeneration of nerve fibers, myelin sheaths and axons (Fig. 5G–K).

### The implantation of 3D-printed collagen/chitosan/HUCMSCs-secretome scaffolds markedly promoted endogenous neuronal differentiation and synapse formation after TBI

**Figure 6.**
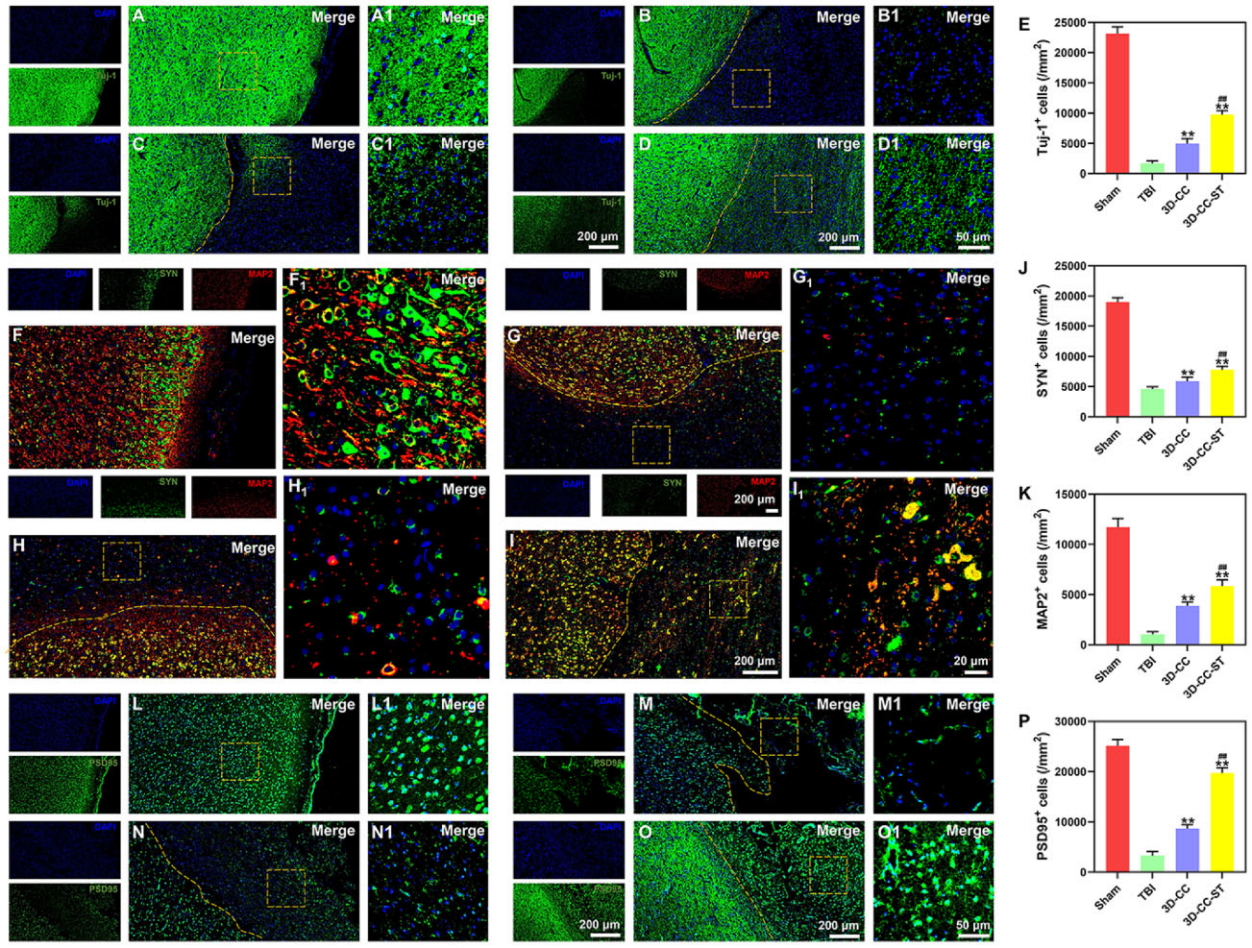
Endogenous neuronal differentiation and synapse formation in the four groups at 6 months after TBI. (**A**–**D1**) Typical images of tuj-1 (green). (**E**) Quantification of tuj-1-positive cells in the peri-injured area after TBI. (**F**–**I1**) Typical images of SYN (green) and MAP2 (red). Quantification of SYN (**J**) and MAP2 (**K**)-positive cells in the peri-injured area after TBI. SYN (green)- and MAP2 (red)-positive cells in the 3D-CC-ST group were increased significantly compared with those in the TBI group and the 3D-CC group. (**L**–**O**) Typical images of PSD95. (**P**) Quantification of PSD95-positive cells in the peri-injured area after TBI. **P <* *0.01 vs TBI. ^##^*P *<* *0.01 vs 3D-CC.

Tuj-1 and MAP2 are markers for early neurons and mature neurons, respectively [44]. We used SYN and PSD95 as indicators of synapses [33, 35, 42]. Endogenous neuronal differentiation of recruited cells is beneficial to axonal regeneration and synaptic formation [45]. At 6 months after surgery, canines implanted with scaffolds had more Tuj-1- and MAP2-positive cells than those without treatment (*P *<* *0.01), and the 3D-CC-ST group showed the most Tuj-1- and MAP2-positive cells (*P *<* *0.01) (Fig. 6A–E, F–I1 and K), which indicated *in vivo* neuronal differentiation induced by 3D-printed collagen/chitosan/HUCMSCs-secretome scaffolds. At 6 months after surgery, in the 3D-CC-ST group, a large number of SYN and PSD95 synapse-like cells were observed in the lesion area, while these cells were relatively few in the TBI and 3D-CC groups (Fig. 6F–J and L–P), suggesting that the implantation of 3D-printed collagen/chitosan/HUCMSCs-secretome scaffolds was beneficial for promoting synapse formation.

### Implanting 3D printed-collagen/chitosan/HUCMSCs-secretome scaffolds reduced cell apoptosis and regulated the level of systemic inflammatory factors after TBI

**Figure 7.**
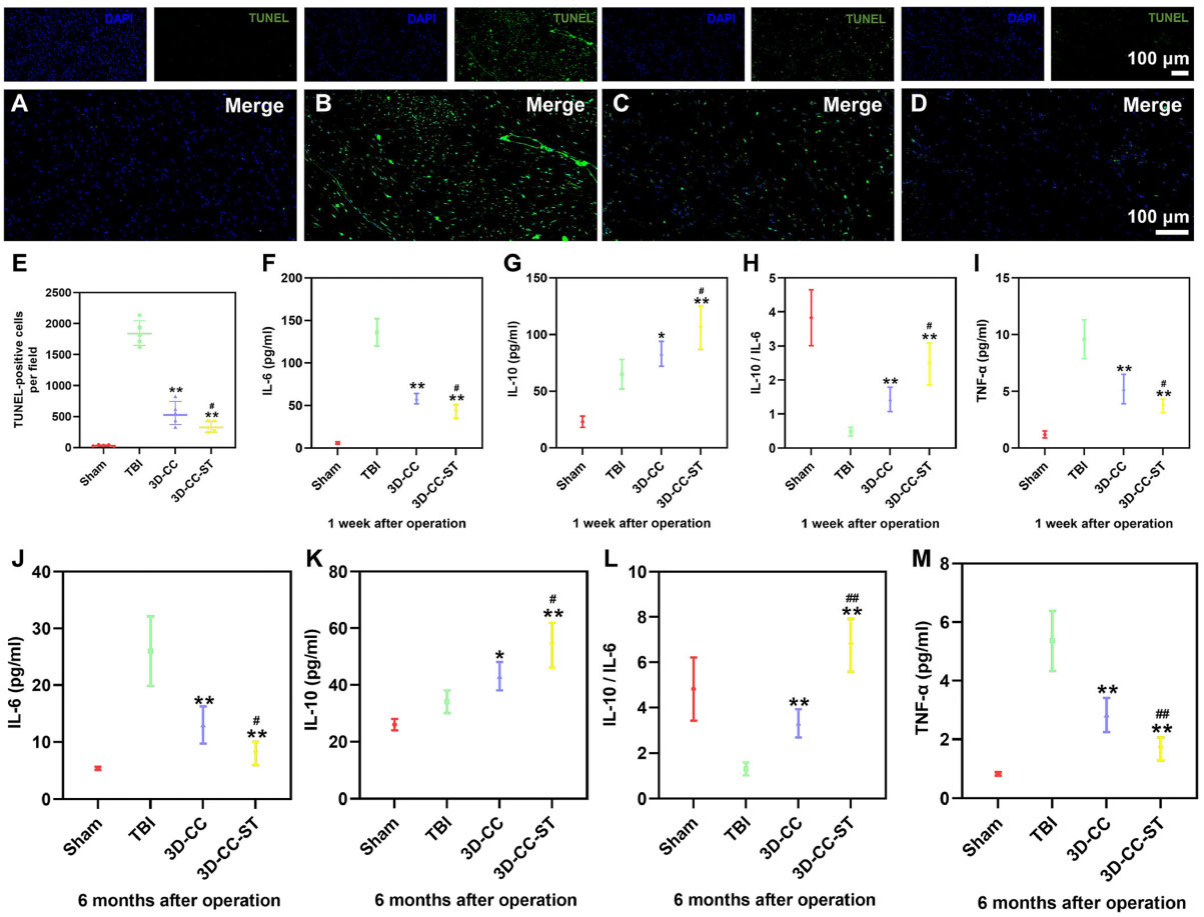
TUNEL Staining of brain tissue at 6 months after TBI and measurement of plasma inflammatory factors at 1 week and 6 months after TBI. Apoptosis in neuronal cells. TUNEL immunostaining in the injured area for the four groups (**A**–**D**). Quantification of TUNEL-positive cells (**E**). Investigation of inflammatory factors at the acute stage (**F**–**I**) and chronic stage (**J**–**M**) in the peri-injured tissue. The expression of IL-6 (F, J) and TNF-α (I, M) was significantly increased in the 3D-CC-ST group at 1 week and 6 months post-surgery compared with that in the TBI group and the 3D-CC group at 1 week and 6 months post-surgery, whereas the expression of IL-10 (**G**, **K**) and IL-10/IL-6 (**H**, **L**) was significantly increased in the 3D-CC-ST group at 1 week and 6 months post-surgery compared with that in the TBI group and the 3D-CC group at 1 week and 6 months post-surgery. *P <* *0.05, **P <* *0.01 vs TBI; ^#^*P *<* *0.05, ^##^*P *<* *0.01 vs 3D-CC.

We further investigated whether the implantation of 3D-CC-ST could ameliorate cell survival and apoptosis by TUNEL staining at 6 months after TBI. Fewer TUNEL-positive cells were observed in the 3D-CC-ST group than in the TBI group and the 3D-CC group (*P *<* *0.05) (Fig. 7A–E), suggesting that implanting 3D-printed collagen/chitosan/HUCMSCs-secretome scaffolds inhibited nerve apoptosis after TBI. To test whether the expression of TNF-α, IL-6, IL-10 and IL-6/IL-10 might be caused by 3D-printed collagen/chitosan/HUCMSCs-secretome scaffolds, the expression was analyzed using ELISA at 1 week and 6 months after the operation (Fig. 7F–M). At 1 week after the operation, compared with the TBI group and the 3D-CC group, the implantation of 3D-CC-ST significantly restrained the expression of TNF-α and IL-6 and augmented the expression of IL-10 (Fig. 7F–I). A similar significant difference was also detected at 6 months after the operation (Fig. 7J–M).

## Discussion

Brain tissue engineering has gradually gained continuous attention due to an increasing number of patients suffering from brain injury, with more than 50 million TBI patients occurring every year globally [5, 46–48]. Management strategies for TBI involve both the treatment of the primary injury and the prevention and treatment of secondary injury. Given the unclear understanding of heterogeneity and complexity, few clinical treatment successes have been described in the last few years. Along with the advances in understanding stem cells, it was ideal for pluripotent stem cells to play a major role in personalized cells because of their ability to differentiate into any cell type of the human body [49, 50]. MSCs with multiple functions in tissue engineering have been regarded as an appropriate candidate to treat TBI. Moreover, considering the painless process for harvesting, lack of ethical problems, low antigenicity, and differentiation ability, HUCMSCs have become a priority in recent years [51]. MSCs are usually characterized by the expression of the surface markers CD73, CD90 and CD105 and are negative for CD11b, CD14, CD19, CD34 and HLADR [52]. Our study showed that MSCs extracted from umbilical cords, which were identified by surface markers, had a high proliferative capability on 3D-printed scaffolds. Currently, it is well known that the paracrine action of MSCs is mainly dependent on their secretion of trophic factors and cytokines [53], which can be classified into five main categories based on their effects: angiogenic, neurogenic, neuroprotective, synaptic and inhibition of scarring [54]. Thus, instead of a cell-based study, HUCMSC-ST, which induced paracrine factors, exosomes and microvesicles, was applied in the treatment.

An emerging number of tissue engineering strategies have been applied to tackle the challenges of biomaterial medicine. It is generally accepted that collagen- and chitosan-based scaffolds, with increased porosity by the effect of compounds on mechanical strength, could offer a microenvironment for essential cell–cell and cell–environment interactions to facilitate tissue regeneration after injury [34, 55]. A variety of materials have been processed into scaffolds with 3D printing technology because pore size is a key point of this scaffold for a suitable microenvironment. Additionally, it has the best degradability and biocompatibility properties among the different mass ratios of 3D-CC-ST. 3D-CC-ST with a mass ratio of 1:8 was the most optional scaffold in the current study. As the collagen/chitosan ratio decreased, in our study, the degradation time displayed a longer time trend. An ideal condition was that the appropriate degradation rate of the desired scaffold should match the tissue repair process [56].

Prior to 3D printing, the collagen, chitosan and secretome derived from HUCMSCs were mixed. On the basis of the above 3D-CC scaffold, a novel strategy to improve the therapeutic effects of ST was to incorporate ST into this scaffold at a low temperature to form a 3D scaffold-incorporated ST (3D-CC-ST), which guaranteed good biocompatibility, a regulatable mechanical modulus and a homogenous distribution of ST. Because of this, the complexes could release ST to the preinjured areas in a sustained manner. Meanwhile, we hope that HUCMSC-ST encapsulated in the 3D-printed scaffold could increase the favorable functions of this scaffold. SEM and HE showed that the porous structure of the internal part of the 3D-printed scaffolds played a useful role in supporting both the adhesion and proliferation of HUCMSCs. Significantly increased proliferation and adhesion were observed in the 3D-CC-ST group compared with the 3D-CC group.

Comprehensive reporting of neurological scores in TBI literature has been described, as there are many outcome measures that can be assessed by subjective priority, leading to evitable bias [57, 58]. MEP revealed that 3D-CC-ST treatment could obviously address the questions of neurological dysfunction. Histological staining and immunostaining of injured brain tissue were applied to observe nerve tissue regeneration, based on the anatomical basis of the locomotor function recovery elicited in the groups. Transplantation of 3D-CC-ST promoted axonal regeneration, mainly because 3D-CC-ST provided a new regenerative microenvironment for nerve fibers.

A number of markers have been reported to be involved in the process of brain tissue engineering, such as neuroregeneration and vascular network construction. To further validate the effect of ST, based on 3D-printed scaffolds, on neuroregeneration, we carried out additional experiments on the potential of ST to incorporate it, which could have a favorable effect on neuronal fibers. In our study, the expression of NF and MBP in the 3D-CC-ST group was higher than that in the TBI group and the 3D-CC group. In the case of GAP43, the expression of this marker is initiated in the morula stages and is essential for neural lineage commitment, as shown in canines treated with 3D-CC-ST. Implanting 3D-printed collagen/chitosan/HUCMSCs-secretome scaffolds significantly facilitated the regeneration of nerve fibers and axons and enhanced remyelination after TBI. In our study, although extensive axonal outgrowth stained by SYN and MAP2 coexpression could be seen in the injury site in both treatment groups, more newborn nerve fibers were found to extend into the lesion site in the 3D-CC-ST group. In addition, we found that 3D-CC-ST reduced the larger number of dead or dying cells caused by TBI and the hostile environment at the injured site compared with the 3D-CC group. The implantation of 3D-printed collagen/chitosan/HUCMSCs-secretome scaffolds markedly promoted endogenous neuronal differentiation and synapse formation after TBI.

Previous studies suggested that inflammation could be the key point of TBI. We focused on the expression levels of proinflammatory factors (such as IL-6 and TNF-α) and anti-inflammatory factor (such as IL-10) in the brain tissue surrounding the injured area [59–61]. The balance between inflammation and anti-inflammation can be measured by the levels and ratios of IL-10 and IL-6 [42]. The decrease in IL-6 and TNF-α and the increase in IL-10 occurred in the 3D-CC-ST group, demonstrating that 3D-CC-ST reduces inflammation in the acute and chronic TBI stages. These results may be related to the anti-inflammatory effect of the HUCMSCs-secretome. Implanting 3D printed-collagen/chitosan/HUCMSCs-secretome scaffolds reduced cell apoptosis and regulated the level of systemic inflammatory factors after TBI.

## Conclusions

The 3D-printed collagen/chitosan/secretome derived from HUCMSCs scaffolds increased the adhesion and proliferation of seeded HUCMSCs. Low-temperature extrusion 3D-printed collagen/chitosan/secretome derived from HUCMSCs scaffolds could promote efficient neural network reconstruction and locomotor function recovery in canines with TBI.

## Funding

This work was supported by the National Major Scientific and Technological Special Project for Significant New Drugs Development (2015ZX09102010).

*Conflicts of interest statement. None declared*.
